# Impacting Health Behaviors of Older Adults Through a Volunteer-Led Health Literacy Program

**DOI:** 10.1093/geroni/igaa057.2853

**Published:** 2020-12-16

**Authors:** Tara Cortes, Liz Seidel, Cinnamon St John

**Affiliations:** 1 New York University, New York, New York, United States; 2 NYU Rory Meyers College of Nursing, New York, New York, United States; 3 Hartford Institute for Geriatric Nursing, New York, New York, United States

## Abstract

A community based, peer volunteer-driven educational program was developed to empower older adults to improve health behaviors through health literacy. A community needs assessment determined the curricula which included classes on chronic disease management and issues impacting older adult health. Volunteers were recruited and trained on the curricula by health educators using a train-the-trainer module. 173 volunteers convened 302 workshops, and trained 2,065 unique older adults who account for 5000 attendees. Using a pretest posttest design, an outcome evaluation found statistically significant increases in knowledge among older adult workshop attendees on 6 of the 7 health topics (p<.01). Eighty-three percent of attendees reported positive health behavior changes at 3-month follow-up. The most commonly cited changes were increased physical activity and improved nutrition. Program challenges included volunteer scheduling and retention. Building a sustainable community-based, peer volunteer-driven health education program for older adults will be discussed.

